# Bio-Sourced, High-Performance Carbon Fiber Reinforced Itaconic Acid-Based Epoxy Composites with High Hygrothermal Stability and Durability

**DOI:** 10.3390/polym16121649

**Published:** 2024-06-11

**Authors:** Kaixuan Xiao, Yuan Fang, Zhaodi Wang, Nannan Ni, Ziqian Liu, Soochan Kim, Zongfu An, Zhiyi Lyu, Yahong Xu, Xin Yang

**Affiliations:** 1 College of Materials Science and Engineering, Nanjing Tech University, Nanjing 211816, China; 202161203303@njtech.edu.cn (K.X.); 202261103096@njtech.edu.cn (Y.F.); 201910006628@njtech.edu.cn (Z.W.); 202010006705@njtech.edu.cn (N.N.); 2 Yangtze River Delta Carbon Fiber and Composites Innovation Center, Changzhou 213000, China; liuziqian@ccicyd.com; 3 School of Chemical Engineering, Sungkyunkwan University, Suwon 16419, Republic of Korea; sc0916@skku.edu (S.K.); zfann@skku.edu (Z.A.); 4 Department of Physics, Sungkyunkwan University, 2066, Seobu-ro, Jangan-gu, Suwon 16419, Republic of Korea; louislv@skku.edu

**Keywords:** sustainable composite materials, itaconic acid epoxy resin, carbon fiber reinforced composites, hygrothermal aging

## Abstract

Thermosetting polymers and composites are a class of high-performance materials with significant industrial applications. However, the widespread use of thermosets and their composites generates large quantities of waste and leads to serious economic and environmental problems, there is a critical need in the elaboration of sustainable composite materials. Here, we propose a method to prepare sustainable carbon fiber reinforced composites with different degrees of greenness by blending environmentally friendly EIA with DGEBA in different ratios, and the properties compared with a well-known commercial petroleum-based epoxy resin. The prepared carbon fiber reinforced polymer (CFRP) composites with different degrees of greenness had excellent dimensional stability under extreme hygrothermal aging. After aging, the green CFRP composite T700/EIA-30 has higher strength and performance retention than that of petroleum-based CFRP composites. The higher hygrothermal stability and durability of EIA-based epoxy resins as compared with BPA-based epoxy resins demonstrated significant evidence to design and develop a novel bio-based epoxy resin with high performance to substitute the petroleum-based epoxy resin.

## 1. Introduction

Epoxy resin (EP) is a thermoset resin with several advantages such as optimal properties, chemical stability, and electrical insulation properties. It is widely used in building materials, aerospace, coating adhesives, and polymer matrix composites [1,2,3,4]. In many high-tech fields, such as carbon fiber reinforced polymer (CFRP) composites, epoxy resins are a major component. However, the widespread use of EP and CFRP generates large quantities of waste and leads to serious economic and environmental problems. Currently, the global production of epoxy resins is expected to grow from 3.55 million tons in 2024 to 4.20 million tons in 2029, at a CAGR (Compound annual growth rate) of 3.41% during the forecast period (2023–2028), in which bisphenol A (BPA) epoxy resins dominate the market [5]. The dominant BPA epoxy resins in the market generate fossilized pollution during the production process. There is limited use of BPA epoxy resin worldwide due to its biotoxicity [6], which requires research and development of environmentally friendly green epoxy resins together with their composites.

It is commonly accepted that green resin composites are mainly fiber-reinforced resins made from bio-sourced materials. However, plant fibers as natural green reinforcements, are difficult to meet the requirements in performance since their hydrophilicity usually shows negative impact on composite properties [7]. Therefore, more attention has been paid to the development of bio-based epoxy resins. The bio-based epoxy resins that are widely studied and applied at present are mainly vegetable oils [8,9], such as soybean oil and hemp oil; lignin [10,11]; and other bio-based compounds that can be reacted with epoxy hydroxypropyl, such as rosin acid [12,13,14], cardanol [15,16], furan derivatives [17], gallic acid [18,19], isosorbide [20], itaconic acid [21], and other small molecules [22]. In recent years, scientists have developed bio-based feedstocks for epoxy resins, which resulted in a series of bio-based epoxy resins. However, epoxy vegetable oil is not suitable for application in the occasion of high requirements for mechanical properties due to the aliphatic long chain structure. Bio-based compounds possessing rigid groups, such as lignin and rosin acid, have been used to develop bio-based resins with high mechanical and thermal properties. Lignin-based epoxy resins result in slow curing and unstable properties due to the low mobility of large molecular substances and complex structures. The fused rings structure in rosin acid epoxy resin leads to an increase in brittleness [23]. Other bio-based compounds, such as cardanol, furan derivatives, gallic acid and isosorbide, still need to consider their cost, yield, and performance [17,24,25,26].

Among them, the development of itaconic acid epoxy resin is noteworthy. Itaconic acid is an unsaturated dicarboxylic acid usually produced by fermenting carbohydrates such as glucose or starch using aspergillus terreus [27]. It is an abundant and cost-effective raw material and has been selected by the U.S. Department of Energy as one of the twelve most promising bio-based platform chemicals [28,29,30]. To the best of our knowledge, itaconic acid has become an excellent raw material for industries such as synthetic resins, synthetic fibers, plastics, rubber, ion-exchange, surfactants, detergents [31]. Ma et al. obtained an itaconic acid epoxy resin (EIA) by reacting itaconic acid with epichlorohydrin, which was shown to have higher epoxide (0.625) and higher reactivity values than bisphenol A diglycidyl ether (DGEBA) [32]. It was found that the thermal and mechanical properties of the EIA were comparable to or better than those of DGEBA. Ma utilized carboxyl and itaconic acid double bonds to design and synthesize trifunctional itaconic acid epoxy resins (TEIA) with high epoxy value and low viscosity, which also has excellent mechanical property. Ma also used double bonds to achieve flame retardant structure and led to excellent flame retardant properties [21,32,33]. However, there is a lack of rigid groups in the crosslinked network of EIA or TEIA, they may be less resistant to solvents and heating. Liu et al. [34]. synthesized an epoxy network based on itaconic acid-based epoxy monomer, maleic anhydride, and glycerol, which possesses well-controlled degradability when combined with carbon fibers to manufacture composites, yet its lower mechanical properties and Tg limit its application.

The application of itaconic acid epoxy resin in carbon fiber-reinforced composites will have great prospects. Generally, applications of carbon fiber-reinforced composites are subject to aging due to ambient humidity or direct exposure to liquids [35]. It is generally believed that the diffusion of moisture in the resin matrix follows Fickian diffusion [36], where the resin undergoes swelling and the glass transition temperature decreases. It is reported that moisture may be located in an unbound state within the resin network or form hydrogen bonds with the resin network, making it difficult to remove [37,38]. It is also important to note that in carbon fiber reinforced composites, although the fibers do not readily absorb moisture, there are gaps between the fibers and the resin matrix, which may further lead to moisture ingress and reduced performance [39,40]. Temperature is a further factor in the aging of composites, as the resin may undergo network relaxation at high temperatures, which accelerates the ingress of moisture and leading to degradation of composite properties. Other factors could also impact composites performance, such as salinity [41], UV irradiation [42], corrosive environment [43] etc.

As an ester-containing bio-based epoxy resin, itaconic acid epoxy resin has low hygrothermal resistance [44]. In order to substitute the petroleum-based epoxy resin and improve the hygrothermal stability and durability of itaconic acid epoxy resin, we propose a method to prepare sustainable carbon fiber reinforced composites with different degrees of greenness by blending EIA with DGEBA in different ratios. The thermal and mechanical properties of the composites were investigated. The hygrothermal aging properties of the composites were also investigated. This paper used a commercial petroleum-based epoxy resin with DGEBA as the matrix of CFRP as control.

## 2. Materials and Methods

### 2.1. Materials

Dicyandiamide (DICY) was purchased from Taicang Changhe Polymer Co., Ltd. (Suzhou, China). Itaconic acid epoxy resin (EIA) with the epoxy value of 0.52 was purchased from Ningbo Institute of Materials Technology and Engineering (Ningbo, China). Diglycidyl ether bisphenol-A (DGEBA) epoxy resin with the epoxy value of 0.51 was purchased from Baling Petrochemical Co., Ltd. (Yueyang, China). Epoxy resin (E-20) was purchased from Baling Petrochemical Co., Ltd. (Yueyang, China). Replaces Urea (GLOC-500) was purchased from Suzhou Chenyang Polymer Material Co., Ltd. (Suzhou, China). Carbon fiber (T700) was purchased from Toray Inc., Tokyo, Japan. None of these chemicals was further purified before usage.

### 2.2. Preparation of the Cured Epoxy Resin

**Figure 1:**
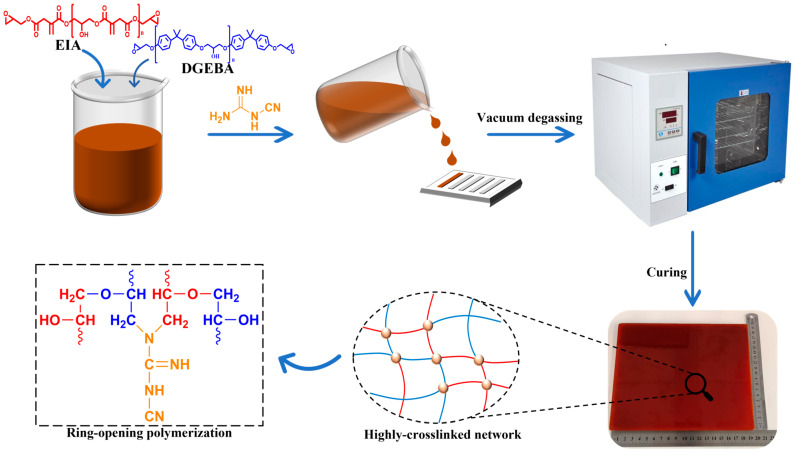
Schematic illustration of preparation of the cured epoxy resin.

First, 20 wt% of E-20 was added to the beaker. After a high-speed stirring at 80 °C for 1 h, different proportions (0 wt%, 30 wt%, 40 wt%, 50 wt%) of EIA and different proportions (70 wt%, 40 wt%, 30 wt%, 20 wt%) of DGEBA were added, and stirred at 1000 r/min for 1 h. After adding 8 wt% of DICY and 2 wt% of GLOC-500, the mixture was heated up to 90 °C and stirred at a high speed of 1500 r/min for 30 min until the DICY and GLOC-500 were homogeneously dispersed in the resin. After vacuuming at 80 °C for half an hour, the product was poured into a polytetrafluoroethylene mold and cured at 80 °C for 1.5 h, 100 °C for 1.5 h, 110 °C for 2 h, and 130 °C for 2 h. Figure 1 and Table 1 show the specific experimental steps and experimental formulations of the products.

### 2.3. Preparation of Carbon Fiber Composite Laminates

CFRP composite laminates of 320 mm × 320 mm were fabricated in an autoclave which allows good control of the curing process. The curing system was heated from room temperature at a heating rate of 3 °C/min to 70 °C for 30 min and then 130 °C for 120 min. This process was subject to a pressure of 3 MPa. The overall thickness of the CFRP laminates was 2 mm, and a total of 16 layers were laid, the other CFRP laminate had a total thickness of 1 mm and were laid with a total of 8 layers. There were 16 layers laid in the thickness direction with the sequence of [0]_16_ and 8 layers laid in the thickness direction with the sequence of [0]_8_, the prepreg average thickness of each layer was 0.13 mm. The density of composites was 1.55 g/cm^3^ and the fiber volume fraction was measured at 60%.

### 2.4. Hygrothermal Aging

Hygrothermal aging tests were conducted on the laminates to observe the moisture absorption behavior of the composites and to determine the weight gain of the composite as a function of time. The size of the moisture absorption specimens was 50 mm × 50 mm × 2 mm, and the water absorption was determined according to ASTM D5229/D5229M standards with a number of five composite specimens in each group [45]. An analytical balance with an accuracy of 0.1 mg was used to measure the weight of each specimen hourly from the first day until the 12th hour, and at regular intervals (every 24 h) from the second day. The experiment was stopped when hygroscopic equilibrium (less than 0.01% change in weight) was reached.

All specimens were pre-treated in a drying oven at 70 °C before starting the experiments until they reached an engineered dry state (less than 0.02% change in weight), and then placed in a thermostatic water bath for hygrothermal aging. Mechanical properties of the composites were tested after 48 h of aging at 80 °C under water immersion. All samples were subsequently weighed to determine weight change. The weight gain was calculated according to

(1) $$M=\frac{M_{w}-M_{d}}{M_{d}}\times 100\%$$

where *M* is the percentage of gained humidity, *M_w_* is the weight of the wet sample, and *M_d_* is the weight of the dry sample.

### 2.5. Characterization

Fourier Transform Infrared Spectroscopy (BRUKER, TENSOR II, Karlsruhe, Germany) was used to investigate the changes in the chemical structure of the resin in the composites before and after hygrothermal aging. FTIR spectra were recorded using a Bruker Tensor II spectrometer with a spectral range of 500 to 4000 cm^−1^ and a resolution of 4 cm^−1^.

The static mechanical properties of the composites were tested using an Instron 3382 material testing machine. Tensile tests were based on ASTM D3039 [46], with each specimen measuring 250 mm × 15 mm × 1 mm, with reinforcing tabs affixed to the ends of each specimen, and with a loading rate of 2 mm/minute for tensile properties, with 6 specimens tested per composite system. Compression test according to ASTM D6641 [47], each specimen size 140 mm × 12 mm × 2 mm, 6 specimens per composite system. Flexural performance test according to ASTM D7264 [48], each specimen size 100 mm × 12.5 mm × 2 mm, flexural performance test loading rate 2 mm/min, 6 specimens per composite system. Interlaminar shear strength (ILSS) test according to ASTM D2344 [49], each specimen size 20 mm × 6 mm × 2 mm, 6 specimens per composite system. The mechanical test samples of composite laminates in this paper were all cut along the 0° direction of the fibers.

Dynamic thermodynamic analysis was carried out by a Dynamic Mechanical Analyzer (TA, DMA Q800, New Castle, DE, USA) in a nitrogen atmosphere. Samples with dimensions of 60 mm × 10 mm × 2 mm were heated from 30 °C to 230 °C at a heating rate of 3 °C/min in three-point bending mode at 1 Hz. 6 samples were tested for each composite system.

Thermogravimetric analysis (TGA) is carried out by means of a thermogravimetric analyzer (NETZSCH, TG 209F, Bavaria, Germany) under a nitrogen atmosphere, 20 mg of powdered solid were heated from 30 °C to 700 °C at a rate of 10 °C per minute.

Damage cross sections of CFRP specimens after flexural performance test were characterized using a TESCAN MIRA4 scanning electron microscope (TESCAN, MIRA4, Brno, Czech Republic). The specimens were gold-plated before SEM observation. The SEM had a working distance of 7.5–15.0 mm, a resolution of 10.0 um, and a voltage of 5 kV.

## 3. Results and Discussion

### 3.1. Moisture Uptake Behavior

**Figure 2:**
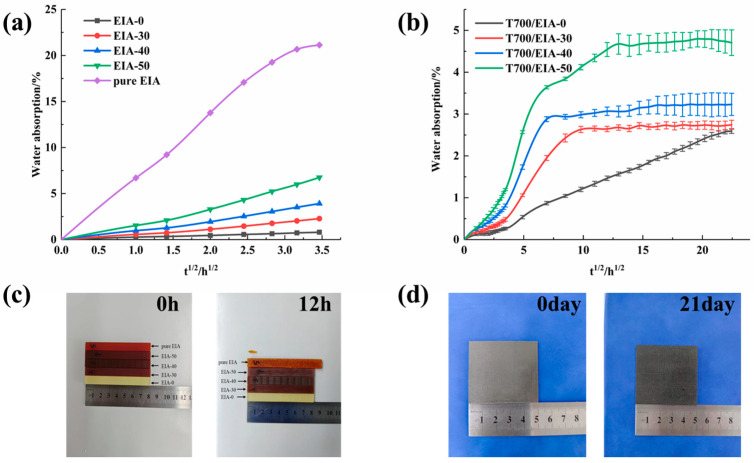
(**a**) Moisture absorption curves for the cured epoxy resin. (**b**) Moisture absorption curves for the composites. (**c**) Representative images of cured epoxy resin after hygrothermal aging. (**d**) Representative images of composites after hygrothermal aging.

Figure 2 shows the water absorption of the epoxy resin and the composite laminates for each system, and since the carbon fibers are essentially non-absorbent, the weight change is caused by the absorption of water by the resin matrix. While EIA lacks rigid groups, increased EIA content leads to increased flexibility of the resin network and easier entry of moisture into the resin. Pure EIA results in decreased dimensional stability of resin in a high-temperature water bath (Figure 2c). Detailed optical pictures are in Figures S1 and S2. However, this phenomenon is not a chemical degradation of the resin since there was no change in the FTIR spectra of the samples before and after hygrothermal aging (Figure S3).

**Figure 3:**
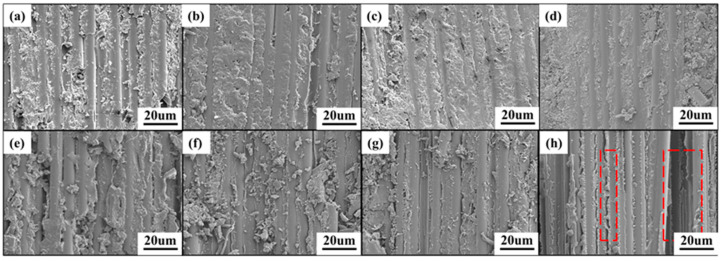
SEM images of the unaged and the aged specimens (**a**) T700/EIA-0 unaged, (**b**) T700/EIA-0 aged, (**c**) T700/EIA-30 unaged, (**d**) T700/EIA-30 aged, (**e**) T700/EIA-40 unaged, (**f**) T700/EIA-40 aged (**g**) T700/EIA-50 unaged, (**h**) T700/EIA-50 aged.

As shown in Figure 2b, for bio-based composite laminates, the water uptake seems to follow a two-stage diffusion response, with the first stage showing a roughly linear relationship between the weight change and t^1/2^, and the diffusion of water following Fickian responses [50]. Whereas, over a longer period, the slow increase in water absorption and equilibrium is reached. As shown in Figure 3, the main reason for the moisture absorption of the resin matrix is the presence of microcracks and the pores on the surface, water can easily enter the composite through these pores, so the water diffusion rate is very fast at the beginning. With longer testing time, the rate of moisture diffusion becomes lower and reaches equilibrium due to the relaxation of the glassy epoxy network [51] and the filling of voids and debonding zones with water by wicking. In Figure 3, the resin in the fiber surface are significantly reduced after hygrothermal aging, but the fibers are still bonded tightly besides the T700/EIA-50. This is because of the higher content of EIA in the T700/EIA-50, the resin matrix in the high temperature water immersion condition is more likely to absorb water, resulting in obvious cracks between the fibers.

As shown in Figure 2b, the composites exhibit a strong tendency to absorb moisture across all systems. This is attributed to the crosslinked network of dicyandiamide cured resin that contains polar groups such as imine, amide, and tertiary amine. With an increase in EIA content, the composites display a higher initial diffusion rate and absorb more moisture at saturation. This is due to the hydrophilic hydroxyl groups of EIA, which facilitate water diffusion [52].

For T700/EIA-0, the benzene ring’s presence and the hydrogen bonds formed between water molecules and the amide or imine impede water diffusion in petroleum-based resin composites. As a result, the rate of water diffusion is lower, and it takes longer to reach water absorption saturation. Nonetheless, the high density of polar groups in these composites results in a higher hydrophilic water absorption rate.

### 3.2. FT-IR Analysis of Composites before and after Hygrothermal Aging

**Figure 4:**
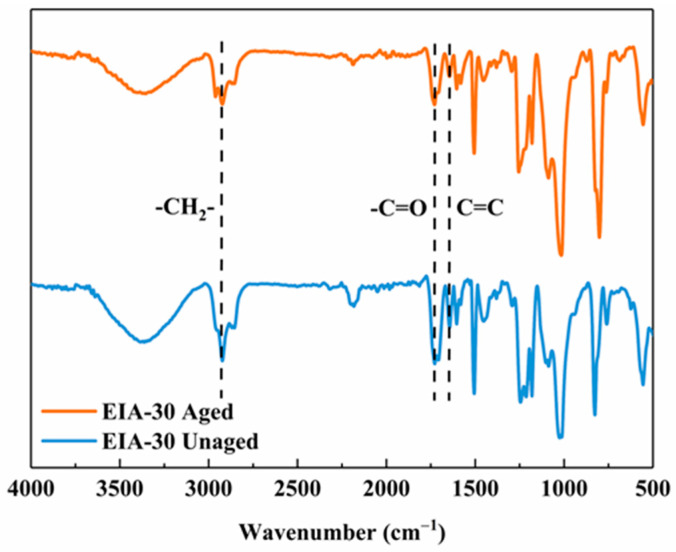
FTIR spectra of unaged EIA-30 resin and aged EIA-30 resin.

Figure 4 illustrates the changes in the infrared spectrogram of the composite EIA-30 before and after hygrothermal aging. the band assigned to the -OH stretching is visible at 3400–3300 cm^−1^ for all the prepared specimens. At 2950 cm^−1^ and 2870 cm^−1^ the asymmetric and symmetric C-H stretch of the methyl and methylene groups were observed. The stretching vibration peak at 1725 cm^−1^ was attributed to -C=O, and the characteristic absorption peaks of C=C was appeared at 1642 cm^−1^, both of which are considered to be the typical structures of EIA [21]. The absorption peak at 2195 cm^−1^ was attributed to -C≡N stretch. It is also possible to observe the characteristic peak at 1604 cm^−1^ and 1506 cm^−1^ were assigned to the benzene ring, and the stretching vibration peaks at 1241 cm^−1^ and 1020 cm^−1^ were assigned to the ether bond. While EIA belongs to aliphatic ether, and the peak of ether bond is reflected at 1090 cm^−1^. There is no increase or decrease in the number of peaks before and after aging, especially the absorption peak at 910 cm^−1^ was attributed to the epoxy group before and after treatment. Indicating that all the specimens have been completely cured during the preparation, and there is no post-curing phenomenon during the aging process [53].

### 3.3. Mechanical Properties of the CFRP Composite

**Figure 5:**
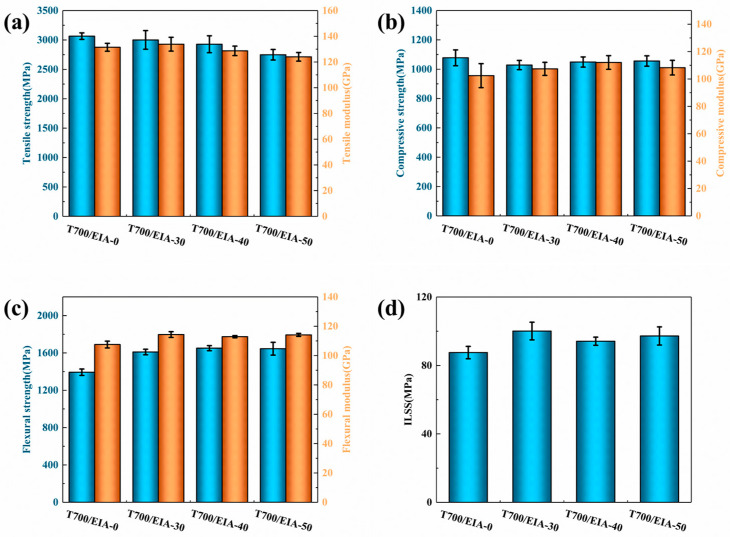
(**a**) Tensile strength and tensile modulus of composites, (**b**) Compressive strength and compressive modulus of composites, (**c**) Flexural strength and flexural modulus of composites, (**d**) Interlaminar shear strength of composites.

Figure 5 shows the basic properties of composite systems with different itaconic acid contents. The detailed data curves can be found in Figures S4–S7. Flexural strength and interlaminar shear strength were significantly increased with the addition of EIA compared to the T700/EIA-0 composite. This is probably attributed to the fact that the resin matrix is more strongly bonded to the carbon fibers, and there is greater resistance to prevent the adjacent layers from undergoing relative displacements. The decline in tensile strength from 3064.70 MPa to 2748.62 MPa with the increase in EIA content is due to the gradual decrease in network rigidity and the ease of displacement and deformation of molecular chain segments under load. There was no significant change in compressive, which may be related to the fracture of fibers unable to contain the stress.

The EIA molecular chain contains an unsaturated double bond structure and contains ester bonds, and exhibits chain flexibility. A limited amount of EIA, the crosslinking network is still dominated by petroleum-based epoxy resin, which will not significantly affect the crosslinking density but can make the crosslinking network toughness increase, and improve the degree of adhesion between the resin and the fiber [54]. While excess EIA is added to the crosslinked network, the relative molecular mass between the crosslinked points is increased after curing and the crosslink density of the system is reduced. The short network chain in the system is prone to become a stress concentration point and would fracture at first so that the material tensile strength was reduced.

**Figure 6:**
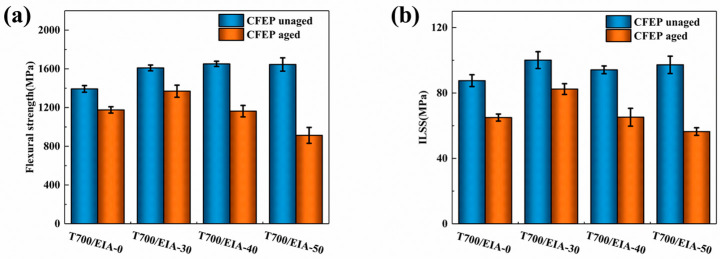
(**a**) Comparing the average flexural strength of the unaged and the aged specimens, (**b**) Comparing the average ILSS of the unaged and the aged specimens.

Figure 6 and Table 2 shows the changes in the properties of the composite system before and after hygrothermal aging. The detailed data curves can be found in Figures S8 and S9. When T700/EIA-30 was immersed in deionized water at 80 °C for 2 days, the bending strength was reduced from 1609.67 MPa to 1369.23 MPa, and the retention rate was 85.06%; the ILSS decreased from 100.11 MPa to 82.42 MPa with a retention rate of 82.33%. As for T700/EIA-0 composites under the same conditions, their flexural strength decreased from 1393.22 MPa to 1175.89 MPa, with a retention rate of 84.40%; ILSS decreased from 86.03 MPa to 64.98 MPa, with a retention rate of 75.53%. When the ratio of EIA to DGEBA is 50/50, the composite has higher strength and performance retention after wet and thermal aging than T700/EIA-0. However, as the percentage of EIA increases, the performance of the composite system decreases more and more after treatment.

The performance degradation of CFRP can be attributed to several factors: temperature, humidity, and load. In the process of moisture absorption, due to the swelling and plasticizing effect of moisture on the resin matrix, the performance of the resin matrix decreases, the composite material would generate new micro-cracks and pores, and the original defects continue to expand. Moisture present within the interface layer of a composite laminate can create internal stresses that diminish interlayer adhesion. In more severe instances, this can cause the resin and fiber to separate, resulting in yield deformation of the fiber and a significant decrease in its strength [55]. The high temperature will accelerate the process of moisture absorption of the laminate so that the composite material will reach the service life and face the scrap. In Table 2, T700/EIA-30 under the condition of high temperature and high humidity can maintain high performance since the EIA improved the adhesion strength of resin and fiber interface, in which preventing moisture from entering. With the increased amount of EIA, the ratio of rigid groups was decreased which led composites have less resistance and make it easier for the system to absorb water, resulting in lower performance.

**Figure 7:**
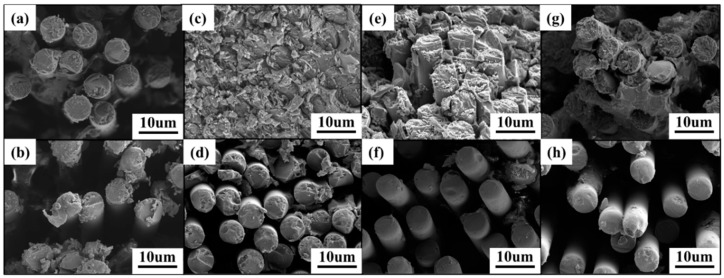
SEM images of the unaged and the aged specimens with fractures (**a**) T700/EIA-0 unaged, (**b**) T700/EIA-0 aged, (**c**) T700/EIA-30 unaged, (**d**) T700/EIA-30 aged, (**e**) T700/EIA-40 unaged, (**f**) T700/EIA-40 aged (**g**) T700/EIA-50 unaged, (**h**) T700/EIA-50 aged.

As shown in the figures (Figure 7a,c,e,g), the fracture morphology of the unaged CFRP specimens was observed by SEM. It can be seen that the fracture surface is uneven, a large amount of resin remains on the fiber surface, and the resin is tightly bonded to the fibers. The main reason for the failure of the composite is the brittle fracture of the fiber and resin. Compare to T700/EIA-0, the adhesion strength of resin and fiber interface was increased after the incorporation of EIA, which improved the mechanical properties of composites. However, with the increased amount of EIA, the mechanical properties of matrix would decrease due to the flexibility of EIA and decreased of crosslink density. Figure 7b,d,f,h show the fracture morphology of CFRP specimens after hygrothermal aging. The attached debris on the fracture surface is obviously reduced, with a neat texture and relatively smooth fiber surface. Resin-fiber debonding occurs and elevated with the increase of EIA content. This is because the EIA molecular chain lacks rigid groups, and the increase in its content resulted in an increase in the water absorption of the composite material.

### 3.4. Thermodynamic Properties of Composites

**Figure 8:**
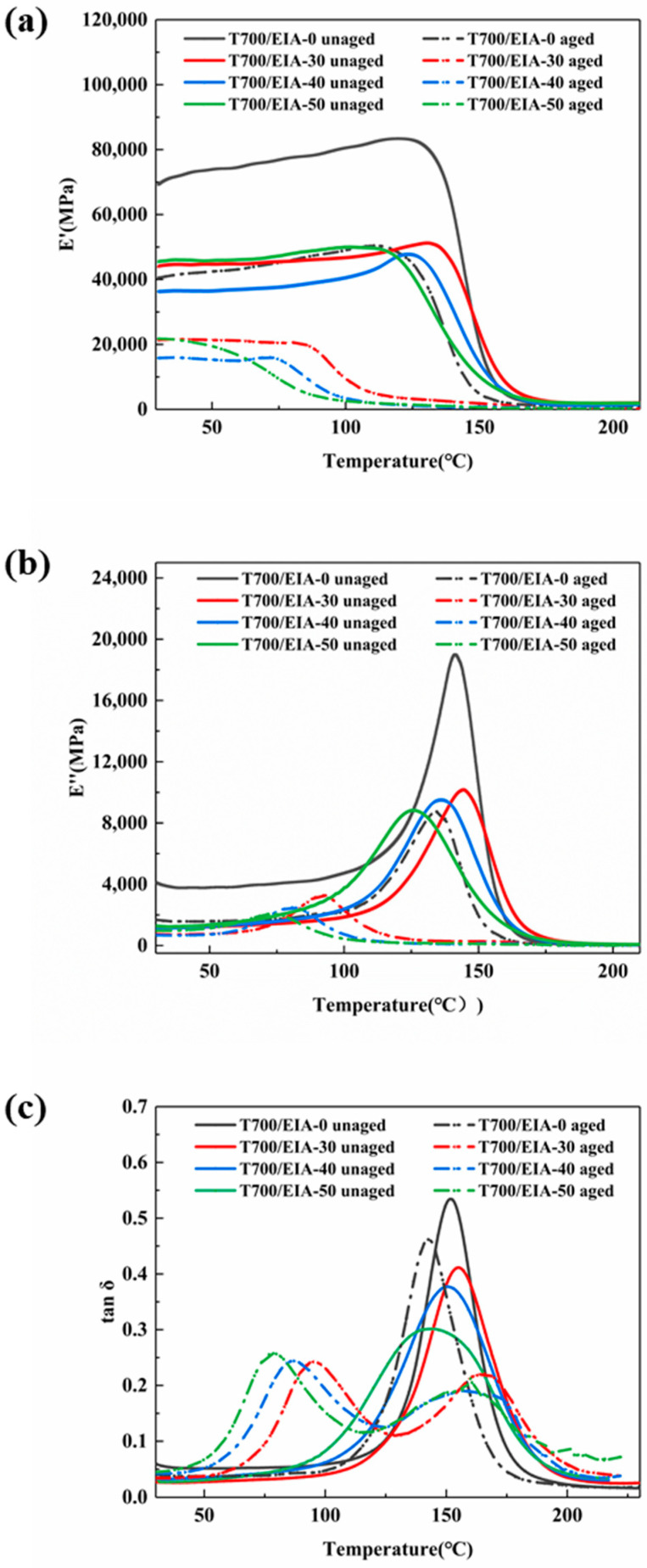
(**a**) Storage modulus, (**b**) loss modulus, and (**c**) loss factor of the CFRP composites with different addition levels (wt%) of EIA.

The dynamic mechanical properties of composite laminates of different systems were investigated using DMA and the results are shown in Figure 8 and Table 3. Figure 8a shows that the initial energy storage modulus (E′) value of T700/EIA-0 composite is greater than that of the composite with EIA addition. As the temperature increases from room temperature, the energy storage modulus retention values of the composites tend to decrease. The higher the EIA content, the lower the temperature at which the energy storage modulus starts to decrease. This result may be due to the thermal movement of the resin chain segments of the composites, while the EIA molecular configuration lacks rigid groups and is worse heat resistant compared to DGEBA. Continuing to increase the temperature, the resin in the composite changes from a glassy state to a rubbery state, allowing the chain segments in the “frozen state” to move freely, thus keeping the energy storage modulus at its sharply decreasing value.

Figure 8b shows the loss modulus (E″) of the composites for each system. In the initial environment, T700/EIA-0 contains a higher loss modulus, which is due to the large proportion of benzene ring content in the resin network structure. At the same time, the addition of EIA reduces the spatial hindrance and chain stiffness, which not only reduces the friction between the polymer chain segments, but also reduces the friction between the resin and the carbon fibers, and less energy is lost. When raised to a certain temperature, the molecular chains are further thawed, the molecular thermal movement is intensified, the friction between the chains as well as the stretching of the molecular chains themselves is intensified, and the loss modulus is increased.

The loss factor is the ratio of loss modulus to energy storage modulus, and Figure 8c shows the loss factor curves of the composites for each resin system. The temperature corresponding to the peak loss factor is the glass transition temperature (T_g_), and the glass transition temperature of T700/EIA-30 (T_g_ = 155.12 °C) is slightly higher than that of T700/EIA-0 (T_g_ = 151.91 °C). As the percentage of EIA content continues to increase, their glass transition temperatures gradually decrease and slightly lower than the T700/EIA-0. This may be due to the fact that low content of EIA increase the cross-linking density of the resin, while when the EIA content is higher, the relative molecular mass between the cross-linking points increases after curing, and the cross-linking density of the system decreases, thus lowering the glass transition temperature of the materials.

The thermomechanical properties of the composites after the hygrothermal aging treatment are also shown in Figure 8. The initial energy storage modulus and initial loss modulus values of the treated composites decrease compared to the untreated values, and the decrease in energy storage modulus is more pronounced for the composites with the addition of EIA. The energy storage modulus retention values start to decrease at lower temperatures, and the higher the percentage of EIA content, the lower the temperature required for the energy storage modulus to start decreasing. The main reason is that the increase of EIA content makes the water absorption rate of the composite material accelerated, and the water enters into the interior of the resin matrix to make the resin swollen, which destroys the network structure of the polymer, and the network is more likely to be thawed out at lower temperatures and transformed from the glassy state to the rubbery state. At the same time, there is a clear relationship between the loss modulus and water absorption, the higher water absorption leads to lower loss modulus. It indicates that the entry of moisture reduces the friction between chain segments, and the collision friction between chain segments may appear as the phenomenon of slipping, which reduces the energy loss. In Figure 8c, the loss factor curves of the composites which contains EIA displayed two peaks after hydrothermal aging, the temperature corresponding to the peak in front is the real glass transition temperature of the resin. The coupling effect of moisture and temperature makes the glass transition temperature shift forward with respect to that of the unaged composites. In contrast, the peak in the back is due to the drying effect in the testing process [56,57].

### 3.5. Thermal Degradation Behavior of the Composites

**Figure 9:**
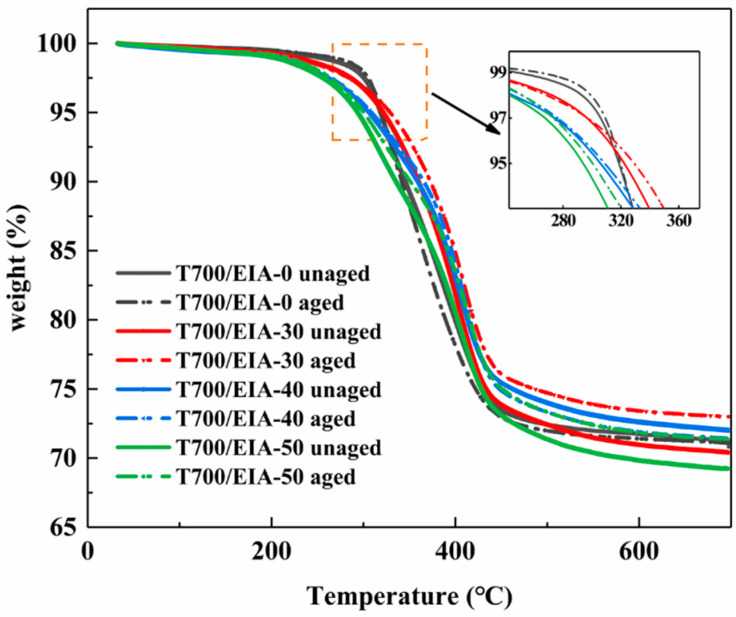
TGA curves of composites with different contents of EIA.

The TGA curves of the composites under nitrogen are shown in Figure 9. The values of initial degradation temperature at 5% weight loss (T_d5%_) are shown in Table 3. The initial decomposition temperature (T_d5%_) of the T700/EIA-30 system is slightly higher than that of the T700/EIA-0 system. When the ratio of EIA in the resin network is higher, the heat resistance of the composites is inferior, because EIA has a large number of easily cleaved ester bonds, it is more susceptible to thermal decomposition than DGEBA. This result is similar to the previous findings of Ma et al. [21]. Another phenomenon is that the initial decomposition temperature (T_d5%_) of each composite system after hygrothermal aging is almost unchanged, suggesting that such treatment does not affect the thermal stability of the material.

## 4. Conclusions

In conclusion, several sustainable carbon fiber reinforced composites with different degrees of greenness by blending EIA with DGEBA in different ratios were prepared. The mechanical and thermodynamic properties of green resin composites were compared with the properties of T700/EIA-0 composites. The effects of hygrothermal aging on the properties of these composites were investigated.

The water absorption tests showed that the prepared specimens had excellent dimensional stability under extreme hygrothermal conditions. Elevated EIA content in the resin leads to faster moisture absorption equilibrium and higher water absorption of the specimens. FTIR spectroscopy showed no evident effect of hygrothermal aging chemical structure of these composites. The mechanical properties of CFRP prepared by each system were comparable. The flexural strengths and ILSS are better than those of T700/EIA-0 in the unaged condition, and the differences in thermomechanical properties were not significant. After 48 h of hygrothermal aging, the water absorption of T700/EIA-30 was higher than that of T700/EIA-0, but the flexural strength, ILSS, and the corresponding retention rate were higher than that of T700/EIA-0, and the thermal stability is comparable to that of T700/EIA-0. It can be seen that the EIA-based epoxy resins as compared with BPA-based epoxy resins demonstrated significant evidence to design and develop a novel bio-based epoxy resin with high performance to substitute the petroleum-based epoxy resin.

## Figures and Tables

**Table 1:** Formulations of epoxy resin with EIA.

Sample	EIA (wt%)	DGEBA (wt%)	E-20 (wt%)	DICY (wt%)	GLOC-500 (wt%)
EIA-0	0	70	20	8	2
EIA-30	30	50	20	8	2
EIA-40	40	40	20	8	2
EIA-50	50	30	20	8	2

**Table 2:** List of mechanical properties of the unaged and the aged specimens.

Sample		Tensile Strength/MPa	Compressive Strength/MPa	Flexural Strength/MPa	Retention Rate of Flexural Strength/%	ILSS/MPa	Retention Rate of ILSS/%
T700/EIA-0	Unaged	3064.70	1077.79	1393.22	100	87.54	100
	aged	-	-	1175.89	84.40	64.98	74.23
T700/EIA-30	Unaged	3000.74	1077.79	1609.67	100	100.11	100
	aged	-	-	1369.23	85.06	82.42	82.33
T700/EIA-40	Unaged	2927.52	1048.90	1651.34	100	94.17	100
	aged	-	-	1162.78	70.41	65.17	69.20
T700/EIA-50	Unaged	2748.62	1055.77	1644.89	100	97.24	100
	aged	-	-	912.53	55.48	56.41	58.01

**Table 3:** List of thermodynamic properties of the unaged and the aged specimens.

Sample		Initial Storage Modulus/GPa	Initial Loss Modulus/GPa	T_g_/°C	T_d5%_/°C
T700/EIA-0	Unaged	69.27	4.12	151.91	318.62
	aged	40.56	1.68	142.57	319.87
T700/EIA-30	Unaged	44.05	1.20	155.12	322.18
	aged	21.59	0.72	94.88	322.62
T700/EIA-40	Unaged	36.29	1.17	150.44	305.63
	aged	15.81	0.68	86.77	308.81
T700/EIA-50	Unaged	45.48	1.28	143.63	293.73
	aged	21.60	0.97	80.98	300.55

## Data Availability

The raw data supporting the conclusions of this article will be made available by the authors on request.

## Supplementary Materials

The following supporting information can be downloaded at: https://www.mdpi.com/article/10.3390/polym16121649/s1, Figure S1: The digital photos of EP samples. (a) EIA-0, (b) EIA-30, (c) EIA-40, (d) EIA-50, (e) Pure EIA; Figure S2: The digital photos of the hygrothermal aging process of EP samples; Figure S3: (a) FTIR spectra of unaged EIA-0 resin and aged EIA-0 resin, (b) FTIR spectra of unaged EIA-40 resin and aged EIA-40 resin, (c) FTIR spectra of unaged EIA-50 resin and aged EIA-50 resin, (d) FTIR spectra of unaged Pure EIA resin and aged Pure EIA resin; Figure S4: Tensile stress-strain curves for CFRP composites. (a) specimens of T700/EIA-0, (b) specimens of T700/EIA-30, (c) specimens of T700/EIA-40, (d) specimens of T700/EIA-50; Figure S5: Compressive stress-strain curves for CFRP composites. (a) specimens of T700/EIA-0, (b) specimens of T700/EIA-30, (c) specimens of T700/EIA-40, (d) specimens of T700/EIA-50; Figure S6: Flexural stress-strain curves for CFRP composites. (a) specimens of T700/EIA-0, (b) specimens of T700/EIA-30, (c) specimens of T700/EIA-40, (d) specimens of T700/EIA-50; Figure S7: ILSS stress-strain curves for CFRP composites. (a) specimens of T700/EIA-0, (b) specimens of T700/EIA-30, (c) specimens of T700/EIA-40, (d) specimens of T700/EIA-50; Figure S8: Flexural stress-strain curves for aged CFRP composites. (a) specimens of T700/EIA-0, (b) specimens of T700/EIA-30, (c) specimens of T700/EIA-40, (d) specimens of T700/EIA-50; Figure S9: ILSS stress-strain curves for aged CFRP composites. (a) specimens of T700/EIA-0, (b) specimens of T700/EIA-30, (c) specimens of T700/EIA-40, (d) specimens of T700/EIA-50.
