# Supplementary material for: Bio-Sourced, High-Performance Carbon Fiber Reinforced Itaconic Acid-Based Epoxy Composites with High Hygrothermal Stability and Durability
Source: Polymers (Basel). 2024 Jun 11;16(12):1649. doi: 10.3390/polym16121649 (PMC11207418; doi:10.3390/polym16121649)
Supplement: Supplementary file 1 [file polymers-16-01649-s001.zip › polymers-3042969-supplementary.pdf]

Supplementary Information

Bio-Sourced, High-Performance Carbon Fiber Reinforced Itaconic Acid-Based Epoxy Composites with High Hygrothermal Stability and Durability

Kaixuan Xiao[1], Yuan Fang[1], Zhaodi Wang[1], Nannan Ni[1], Ziqian Liu[2], Soochan Kim[3], Zongfu An[3], Zhiyi Lyu[4], Yahong Xu[1*], Xin Yang[1*]

[1] College of Materials Science and Engineering, Nanjing Tech University, Nanjing 211816, China

[2] Yangtze River Delta Carbon Fiber and Composites Innovation Center, Changzhou 213000, China

[3] School of Chemical Engineering, Sungkyunkwan University, Suwon, 16419 Republic of Korea

[4] Department of Physics, Sungkyunkwan University, 2066, Seobu-ro, Jangan-gu, Suwon, Gyeonggi-do 16419, Republic of Korea

* Correspondence: 201910006672@njtech.edu.cn (Y.X.); xin12.25@hotmail.com (X.Y.)

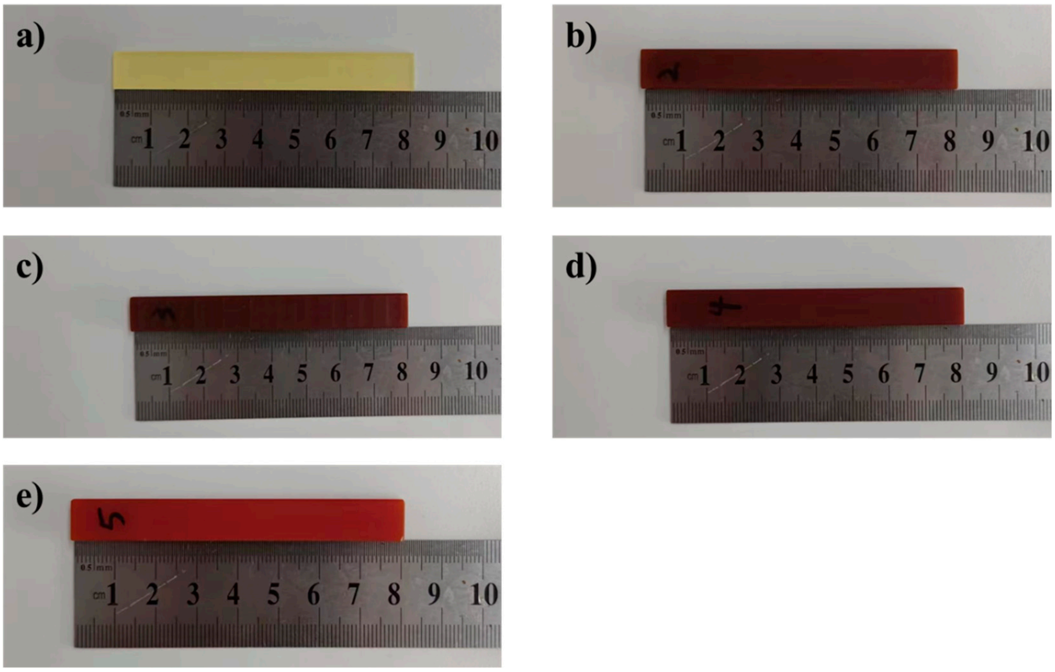

Figure. S1. The digital photos of EP samples. (a) EIA-0, (b) EIA-30, (c) EIA-40, (d) EIA-50, (e) Pure EIA.

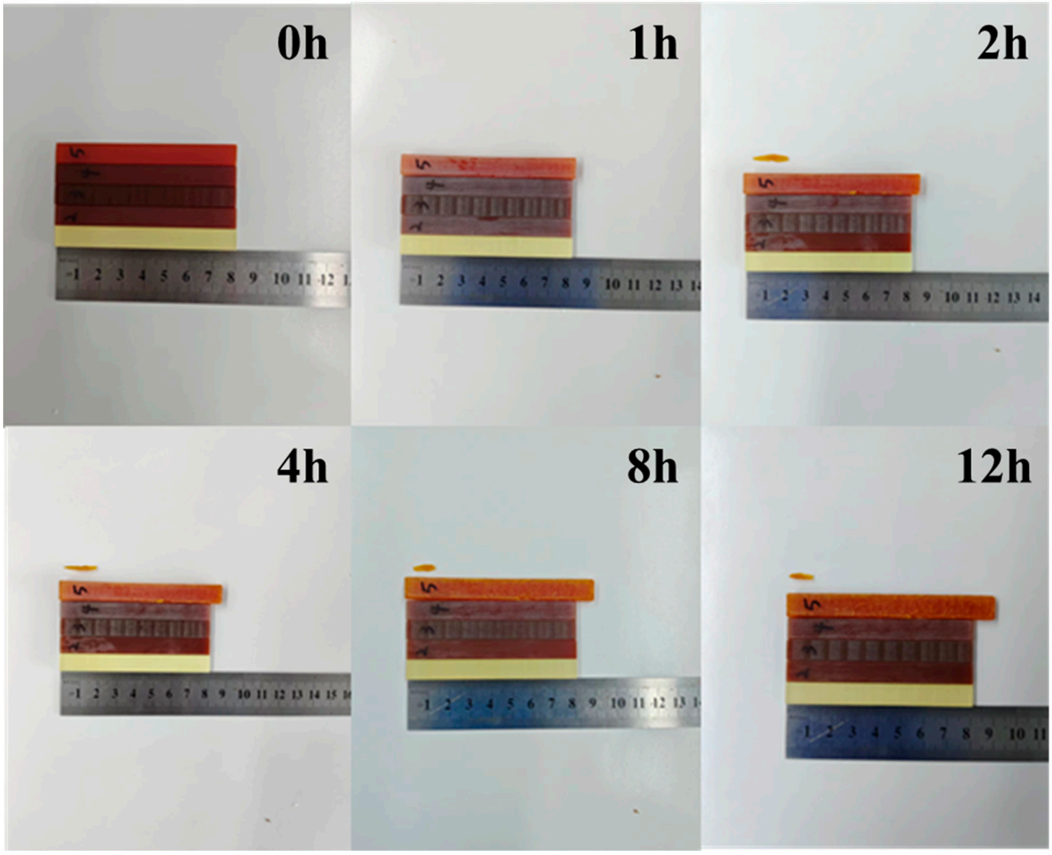

Figure. S2. The digital photos of the hygrothermal aging process of EP samples.

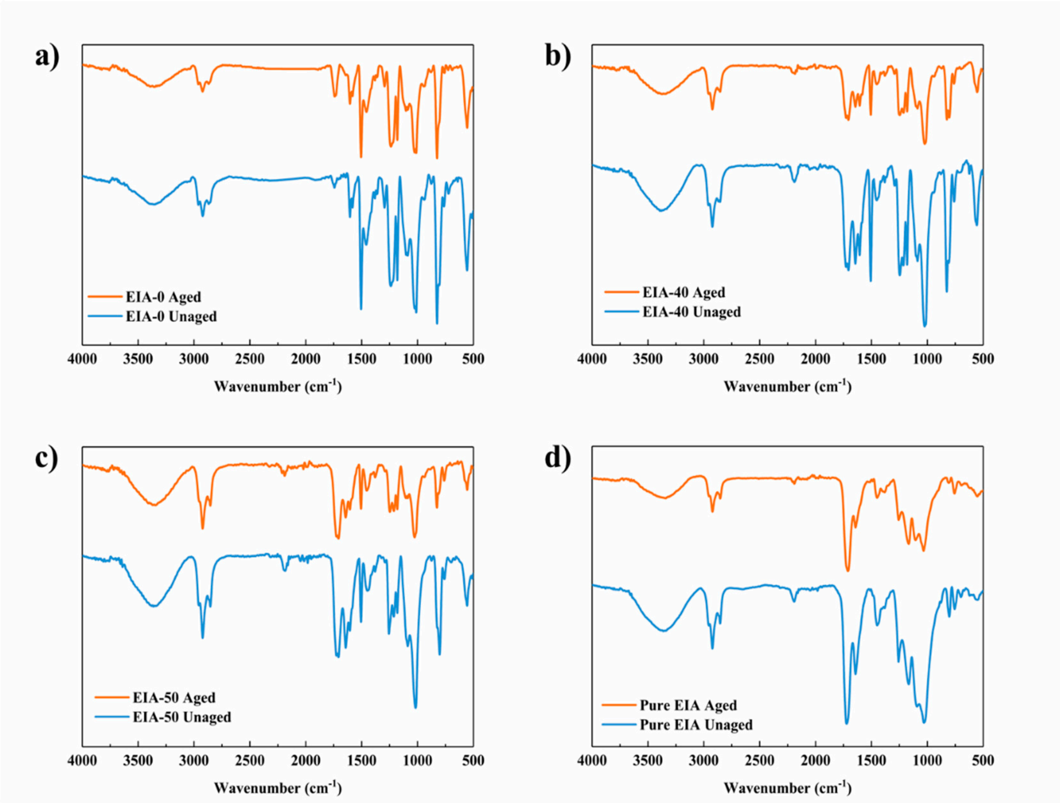

Figure. S3. (a) FTIR spectra of unaged EIA-0 resin and aged EIA-0 resin, (b) FTIR spectra of unaged EIA-40 resin and aged EIA-40 resin, (c) FTIR spectra of unaged EIA-50 resin and aged EIA-50 resin, (d) FTIR spectra of unaged Pure EIA resin and aged Pure EIA resin.

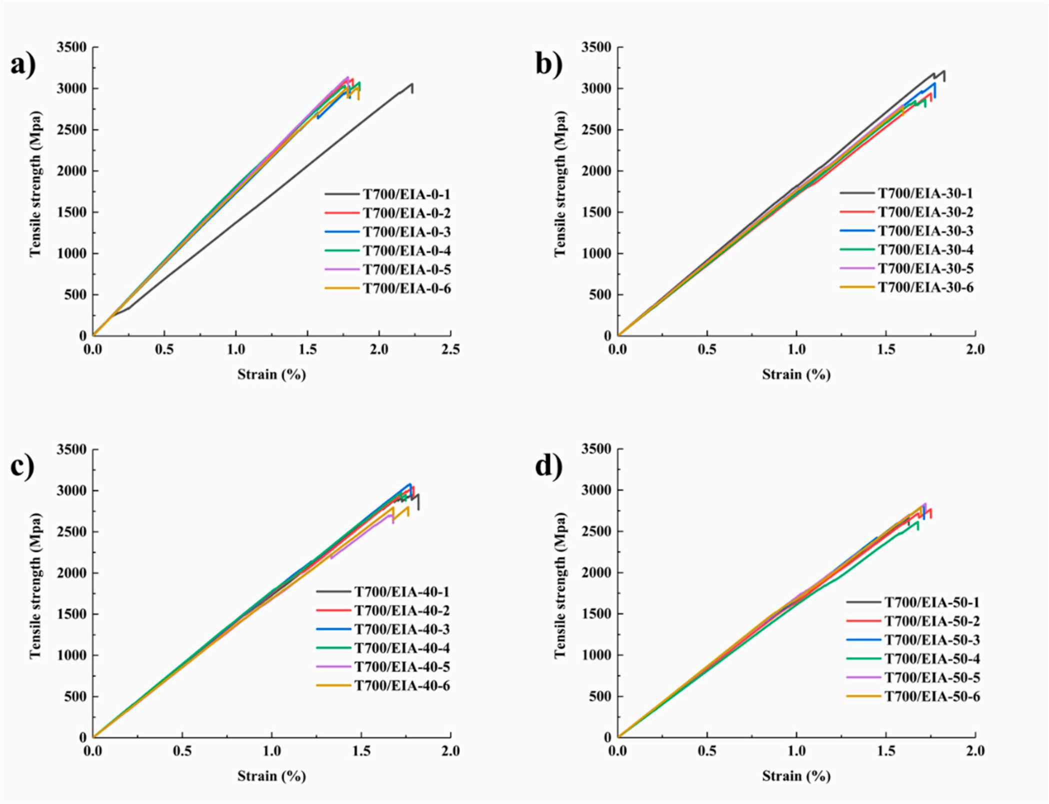

Figure. S4. Tensile stress-strain curves for CFRP composites. (a) specimens of T700/EIA-0, (b) specimens of T700/EIA-30, (c) specimens of T700/EIA-40, (d) specimens of T700/EIA-50.

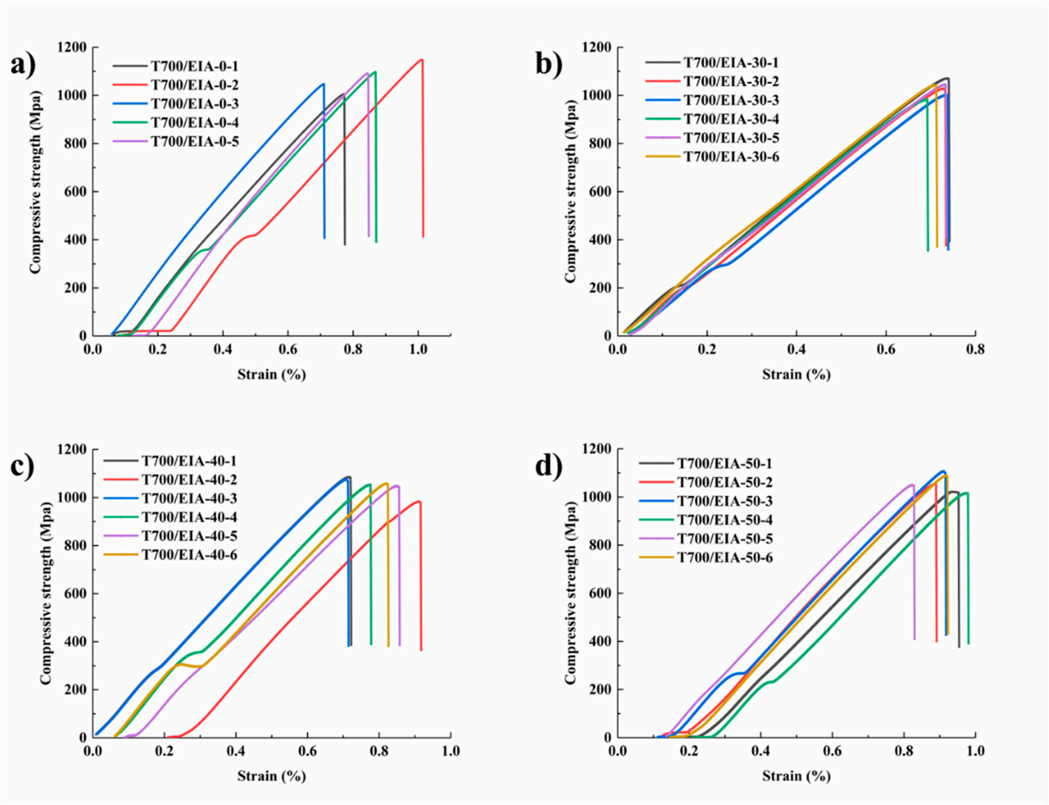

Figure. S5. Compressive stress-strain curves for CFRP composites. (a) specimens of T700/EIA-0, (b) specimens of T700/EIA-30, (c) specimens of T700/EIA-40, (d) specimens of T700/EIA-50.

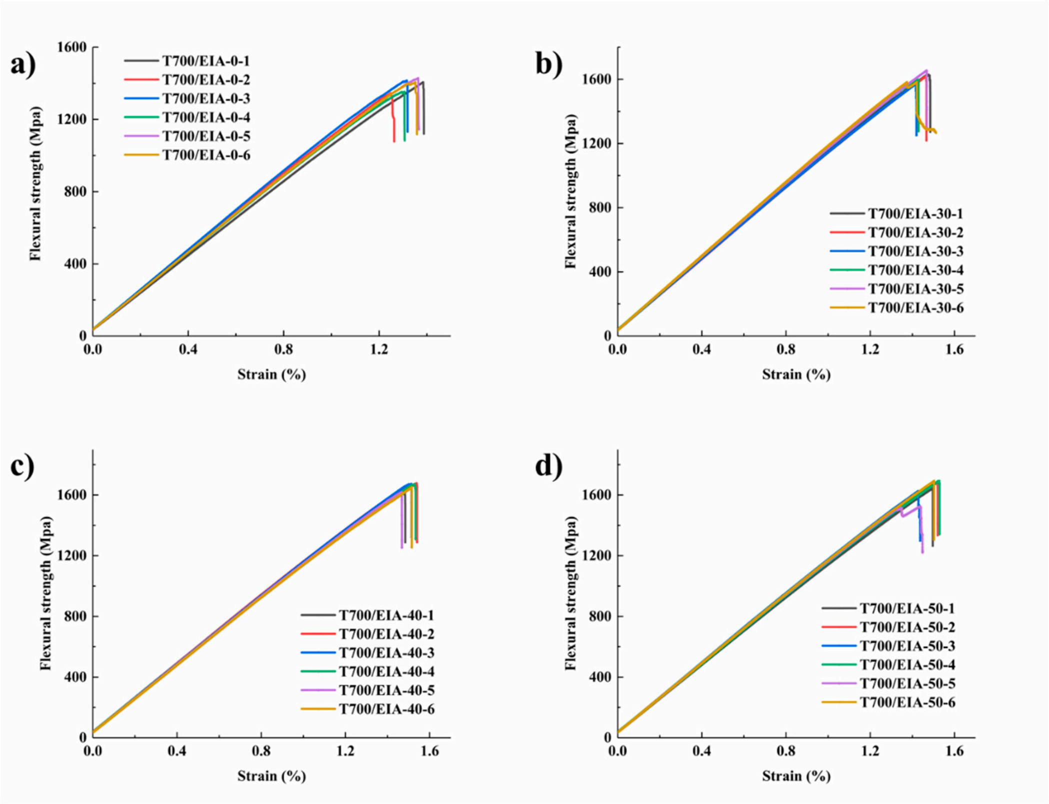

Figure. S6. Flexural stress-strain curves for CFRP composites. (a) specimens of T700/EIA-0, (b) specimens of T700/EIA-30, (c) specimens of T700/EIA-40, (d) specimens of T700/EIA-50.

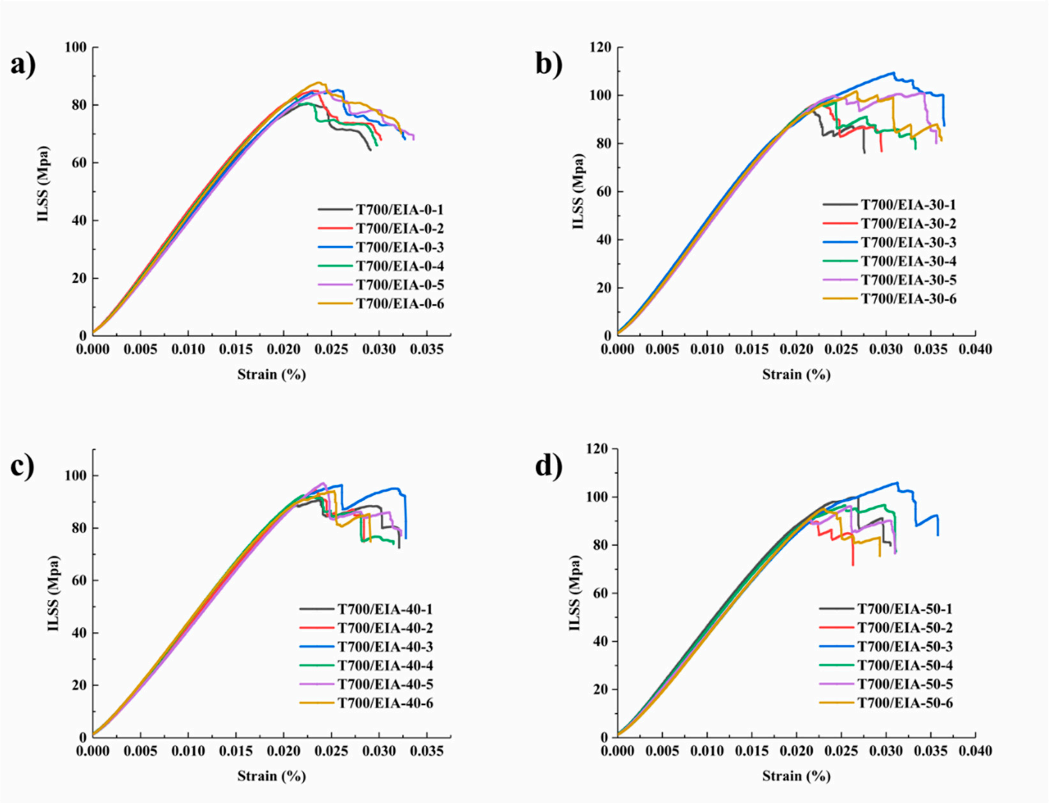

Figure. S7. ILSS stress-strain curves for CFRP composites. (a) specimens of T700/EIA-0, (b) specimens of T700/EIA-30, (c) specimens of T700/EIA-40, (d) specimens of T700/EIA-50.

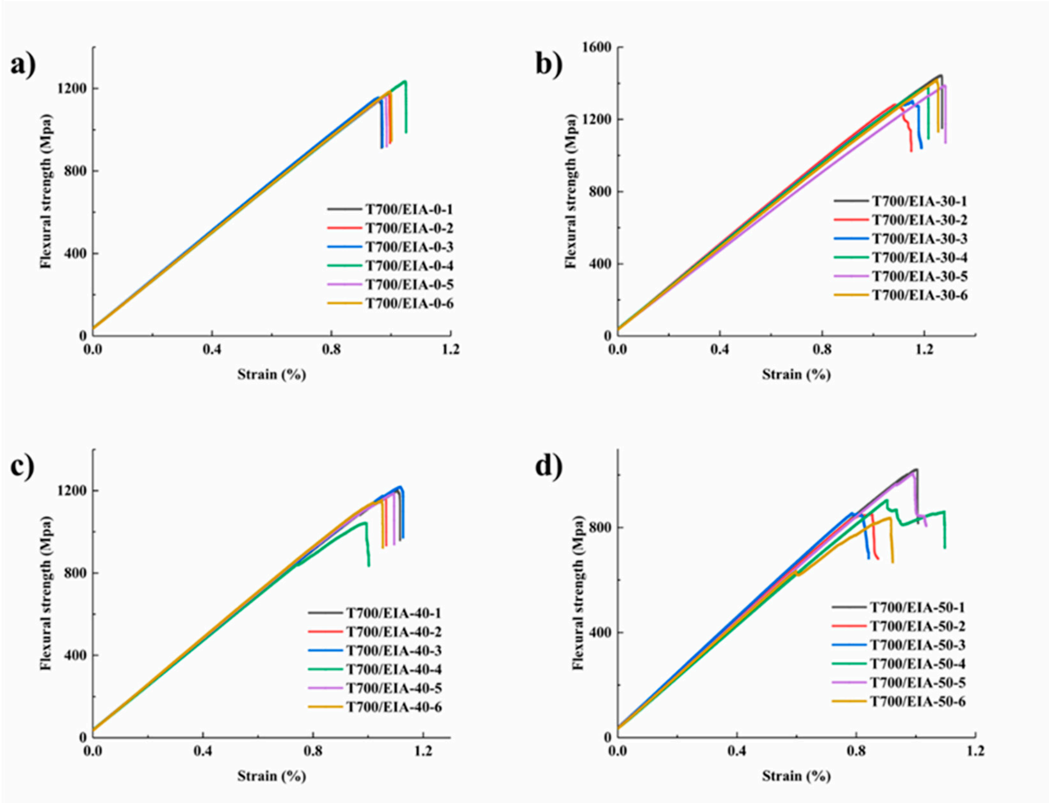

Figure. S8. Flexural stress-strain curves for aged CFRP composites. (a) specimens of T700/EIA-0, (b) specimens of T700/EIA-30, (c) specimens of T700/EIA-40, (d) specimens of T700/EIA-50.

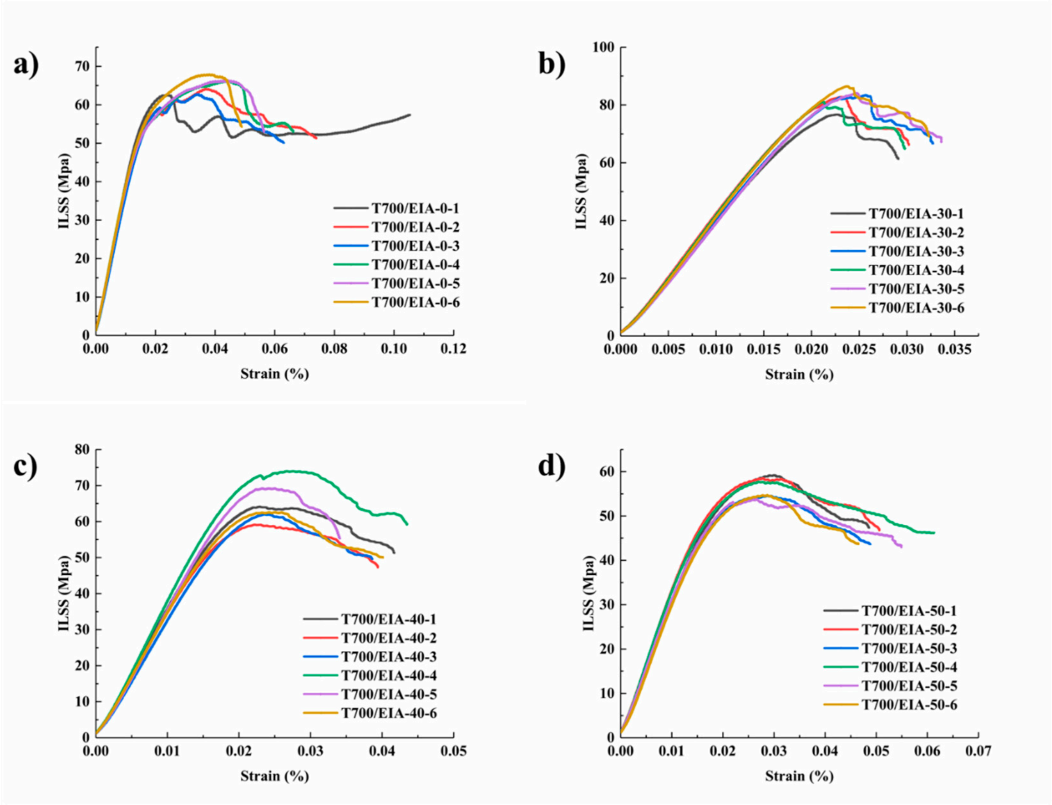

Figure. S9. ILSS stress-strain curves for aged CFRP composites. (a) specimens of T700/EIA-0, (b) specimens of T700/EIA-30, (c) specimens of T700/EIA-40, (d) specimens of T700/EIA-50.
